# Shape and spatial working memory capacities are mostly independent

**DOI:** 10.3389/fpsyg.2015.00581

**Published:** 2015-05-20

**Authors:** Motoyuki Sanada, Koki Ikeda, Toshikazu Hasegawa

**Affiliations:** ^1^Department of Cognitive and Behavioral Sciences, Graduate School of Arts and Science, The University of TokyoTokyo, Japan; ^2^Japan Society for the Promotion of ScienceTokyo, Japan; ^3^Department of Psychology, Chukyo UniversityNagoya, Japan

**Keywords:** working memory capacity, visual working memory, spatial working memory, visuo-spatial sketchpad, dual task

## Abstract

Whether visual working memory (WM) consists of a common storage resource or of multiple subsystems has been a controversial issue. Logie (1995) suggested that it can be divided into visual (for color, shape, objects, etc.) and spatial WM (for location). However, a recent study reported evidence against this hypothesis. Using a dual task paradigm, Wood (2011) showed interference between shape and spatial WM capacities, suggesting that they share a common resource limitation. We re-examined this finding controlling possible confounding factors, including the way to present spatial location cues, task order, and type of WM load to be manipulated. The same pattern of results was successfully reproduced, but only in a highly powered experiment (*N* = 90), and therefore the size of interference was estimated to be quite small (*d* = 0.24). Thus, these data offer a way to reconcile seemingly contradicting previous findings. On the one hand, some part of the storage system is genuinely shared by shape and spatial WM systems, confirming the report of Wood (2011). On the other hand, the amount of the overlap is only minimal, and therefore the two systems should be regarded as mostly independent from each other, supporting the classical visuo-spatial separation hypothesis.

## Introduction

It is well accepted that working memory (WM) is separated into two systems, namely, phonological (the phonological loop) and visual information storages (the visuo-spatial sketchpad; Baddeley and Hitch, 1974). More controversial is the next assumption that the latter can be further divided into two substructures for visual (colors, shapes, object, etc.) and spatial (location) information processing (Logie, 1995), which are usually referred as visual and spatial WM, respectively.

Evidence for this visuo-spatial separation hypothesis has been mixed so far (Luck, 2008). On the one hand, dual task experiments have provided some supporting data. In a typical dual task paradigm, a cognitive task is inserted during the retention interval of a WM task, thus participants have to perform the task and WM maintenance simultaneously. Studies found that the interference between the two tasks became significantly larger when they were related to the same domain (e.g., visual task and visual WM) than when they were related to different domains (Logie and Marchetti, 1991; Tresch et al., 1993; Woodman et al., 2001; Woodman and Luck, 2004). For example, Tresch et al. (1993) found that spatial WM performance was selectively disrupted by a movement discrimination task (i.e., a spatial cognitive task) but not by a color discrimination task (i.e., a feature-based visual cognitive task), whereas the opposite pattern of results was obtained for a shape WM task.

Other studies have suggested that a more nuanced argument might be required for this issue. For example, Wheeler and Treisman (2002) hypothesized that keeping spatial information may not be necessary for maintaining simple features (e.g., “blue” or “triangle”) but critical for conjunctive objects (e.g., “blue triangle”; see, however, Zhang et al., 2012). Furthermore, using a change detection task in which the test display was either exactly the same as the memorized one or differed from it in one item, Jiang et al. (2000) reported that performances of simple feature WM tasks (i.e., color or shape) were impaired when item locations were changed between to-be-remembered and to-be-matched stimuli, even though the spatial information was totally task-irrelevant. Although these studies did not directly test the visuo-spatial WM separation hypothesis, they suggested that the proposed dichotomy might be rather simplistic and further investigations were required.

Wood (2011) tackled this problem by investigating the dual task paradigm again, but now in a thoroughly systematic way. In addition, he investigated the consequences of directly combining two WM tasks. This was a notable attempt, since the majority of the previous studies that had utilized the dual task paradigm had only focused on the interference between a WM and a *cognitive* task (e.g., a movement discrimination task; Tresch et al., 1993), which does not examine the interferences between two WM tasks and therefore might not be a direct test of the visuo-spatial separation hypothesis. In contrast, Wood (2011) combined spatial WM tasks with various types of visual WM tasks with the following basic design. White dots appeared on a computer screen at the beginning of each trial, and participants were requested to remember their locations. Next, items with simple color, shape, or conjunctive features were presented, and participants had to remember their identities, too. After a brief blank, a test array was presented, which could be matched with either the spatial WM locations or the visual WM items that were remembered previously. In most of the experiments, the so-called “single probe” task was adopted to test visual WM, where only one to-be-matched item is presented on the test display. The number of to-be-remembered locations and items were manipulated to examine whether and when interference occurred between the two domains.

Despite his effort for an inclusive examination, the results of Wood (2011) only added further complications to the issue. In Experiment 2, he found that increasing the number of spatial cues did not disrupt the performance of the *color*, but did interfere with the *shape* and* object* (color–shape conjunction) tasks. No previous theories and studies are fully consistent with these new data. Firstly, these data clearly contradict the traditional hypothesis of visuo-spatial WM separation, which would have predicted no interference between visual (including shape) and spatial WM (Tresch et al., 1993; cf. Woodman et al., 2001). Secondly, based on the theory of Wheeler and Treisman (2002), overloading spatial WM would have been predicted to deplete the capacity for spatial information maintenance, and therefore interfere with object but not simple feature (e.g., shape) WM. On the other hand, the data of Jiang et al. (2000) would have suggested that spatial WM load would generally affect the spatial information maintenance and therefore interact with *both* shape and color WM.

What are the causes of these discrepancies? The first possibility is the differences in task designs, especially between the change detection and single probe tasks. Wood (2011) reported that the interference between color (i.e., a feature) and spatial WM was found only in the change detection, but not in the single probe task. He therefore speculated that in the change detection task, but not in the single probe task, spatial configural information is employed to retain not only spatial, but also visual WM including simple features. In accordance with this hypothesis, Jiang et al. (2000) utilized the change detection paradigm and observed impairments *even* in feature WM performance (i.e., color and shape) when the locations of items were changed during a trial, lending some credibility to the argument. This is, however, not sufficient to account for the interference between shape and spatial WM found in Wood (2011), because the interference was detected not only in the change detection, but also in the single probe task.

In order to reconcile the shape-spatial WM interference reported by Wood (2011) with the previous literature, the current study tried to replicate this finding while controlling some possibly confounding factors observed in the original study. Our hypothesis was that these factors might have caused the discrepancy, and therefore a clear conclusion could be obtained if they were fully controlled. The first factor was related to a specific methodological detail Wood adopted, which has already been discussed in some previous studies (Woodman and Luck, 2004; Lecerf and De Ribaupierre, 2005). That is, since multiple white dots appeared simultaneously on the computer screen in Wood (2011), the participants might have encoded them as *a shape* formed by these white dots rather than separate *spatial locations*. If this was the case, the observed shape-spatial interference could be interpreted as having occurred between two shape WM tasks. We examined this possibility in Experiments 1a,b,c. Next, we also examined the effect of task order (Experiment 2) and types of WM load to be manipulated (Experiment 3). These factors were not, or only minimally manipulated in the original study. To foreshadow the results, none of the controls altered the results. Moreover, no statistically significant evidence of between-domain interference was found in any of these experiments, seemingly disconfirming the observations of Wood (2011). Importantly, however, we found a very small, but consistent trend of interference in all experiments regardless of the different settings, suggesting the obtained null results were simply due to under-powered designs. Therefore, we conducted an omnibus test including four of these experiments and found a small, but statistically significant effect. We also conducted another replication following the design of Wood (2011) more precisely (Experiment 4), in which we collected data from 90 participants to sufficiently increase statistical power. A significant effect of interference was observed again, but its effect size remained to be quite small. We concluded that, although there was an overlap between spatial and shape WM processing, the size of this effect was small, and therefore the two systems should be regarded as mostly independent from each other.

## Experiment 1

The purpose of Experiments 1a,b,c was to test if the results of Experiment 2 in Wood (2011) were due to the specific methods that the study adopted for the spatial WM cue presentation or data analysis. Sequential cue presentation was used in Experiments 1a,b, and simultaneous cue presentation in Experiment 1c. We examined the interaction between task type and load manipulation as a measure of interference in all three experiments.

### Experiment 1a

#### Methods

##### Participants

Thirty volunteers (male: 15; female: 15; mean age: 19.93 years, SD: 2.00 years) participated in the experiment. They provided informed consent before commencing the experiment and were compensated monetarily.

##### Stimuli and procedure

All stimuli were presented on a black screen of a 17 inch CRT monitor, and E-prime 2.0 (Psychology Software Tools, Inc., Sharpsburg, PA, USA) was used to program the experiment. The viewing distance was about 60 cm.

At the beginning of a trial, two alphabet letters (white, bold, and 45 point Courier New font) were randomly selected and presented for 1,000 ms at the center of the screen. Participants had to pronounce these letters repeatedly for articulately suppression until they responded to the test array (see below), in order to prevent the spatial and shape stimuli from being verbalized. To confirm if participants correctly followed this instruction, we recorded their voices all through the experiment by a voice recorder. Participants were informed about this recording procedure beforehand. After the letter presentation, the word “Ready” (white, bold, and 45 point Courier New font) appeared for 500 ms at the center of the screen, followed by a 500 ms blank and then a spatial memory array.

**FIGURE 1:**
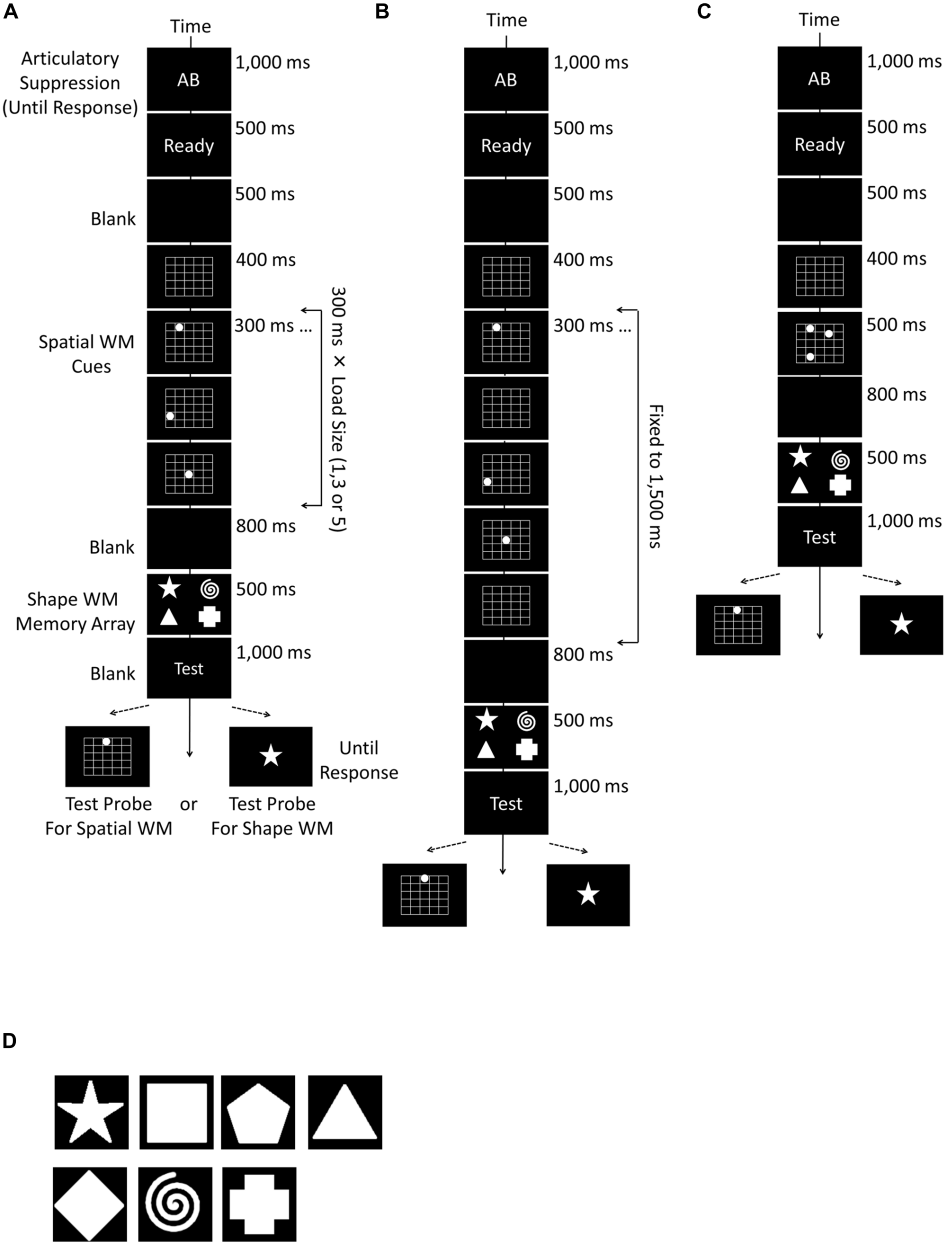
**Schematic diagrams of the experimental procedures used in Experiments 1a (A), 1b (B), and 1c (C)**. In all experiments, a trial consisted of the instruction of articulatory suppression, spatial working memory (WM) cues, first blank, shape WM array, second blank, and the test probe. Spatial WM was tested in the half of trials, and shape WM in the rest of trials. The load size of spatial WM randomly changed from 1, 3, to 5, but that of the shape WM was fixed to 4. The only difference between Experiments 1a,b was the way to present spatial WM cues. The total time to present spatial WM cues changed in accordance with the number of the load in Experiment 1a, whereas fixed to 1,500 ms in Experiment 1b. **(D)** Shows the shape stimuli which were used for the shape WM task in Experiments 1a – c, 2, and 4.

Unlike Wood (2011), the spatial memory array was presented in a sequence. A 5 × 5 grid (width 17.6∘ × length 14.7∘) with white borders appeared for 400 ms at the center of the screen, and consecutively white dots (2.2∘ × 2.2∘) were randomly presented one by one, each for 300 ms, in one of the cells in the grid. The white dots in one trial never appeared in the same cell. There was no interval between dot presentations, thus the entire presentation time changed according to the set size of the memory array; they were 300, 900, and 1,500 ms for the set size 1, 3, and 5, respectively. Participants had to remember all locations, but not the order of presentation. This spatial memory task was followed by a 800 ms blank and the shape memory array (**Figure 1A**).

The shape memory array consisted of four white shapes randomly selected from seven distinguishable items (star, square, pentagon, triangle, diamond, spiral, and cross, see **Figure 1D**), all of which had a size of 3.2∘ × 3.2∘. They remained on the screen for 500 ms. The locations were fixed on the corners of a width 10.1∘ × length 6.1∘ rectangle appearing on the center of the screen. The participants had to remember the shapes but not their locations. After the shape memory array, the word “Test” (white color, bold, 45 point Courier New font) appeared for 1,000 ms, and was followed by the memory test.

Two different versions of WM test were used; that is, one for the spatial and another for shape memory, each occurring with a probability of 50%. Note that participants had to retain both spatial and shape information in all trials, since the selection of test type was totally random. For testing spatial WM, a white dot appeared in one of the 5 × 5 grid cells. In half of trials (i.e., 25% of all trials), the dot appeared at one of the locations where the to-be-memorized items had been presented previously (the same condition), and in one of the remaining locations in the rest of trials (the different condition). For assessing shape WM, a shape was selected from the aforementioned seven shapes (see **Figure 1C**) and presented at the center of the screen. It matched with one of the to-be-memorized shape items in half of the trials (the same condition) but not in the rest (the different condition). In both versions, participants had to answer whether the test item matched with one of the items retained in memory, by pressing the “f” or “j” key on the keyboard (the key-response correspondence was counterbalanced across participants). The test item remained on the screen until the response. A 300 ms blank was inserted as inter-trial interval before the next trial started.

The experiment comprised 30 blocks, each of which contained 12 trials, thus the total number of trials was 360. At the end of each block, accuracy rates of spatial and shape memory test were presented on the monitor. Participants conducted two practice blocks with trial-by-trial accuracy feedback before starting the experiment.

### Results and Discussion

**FIGURE 2:**
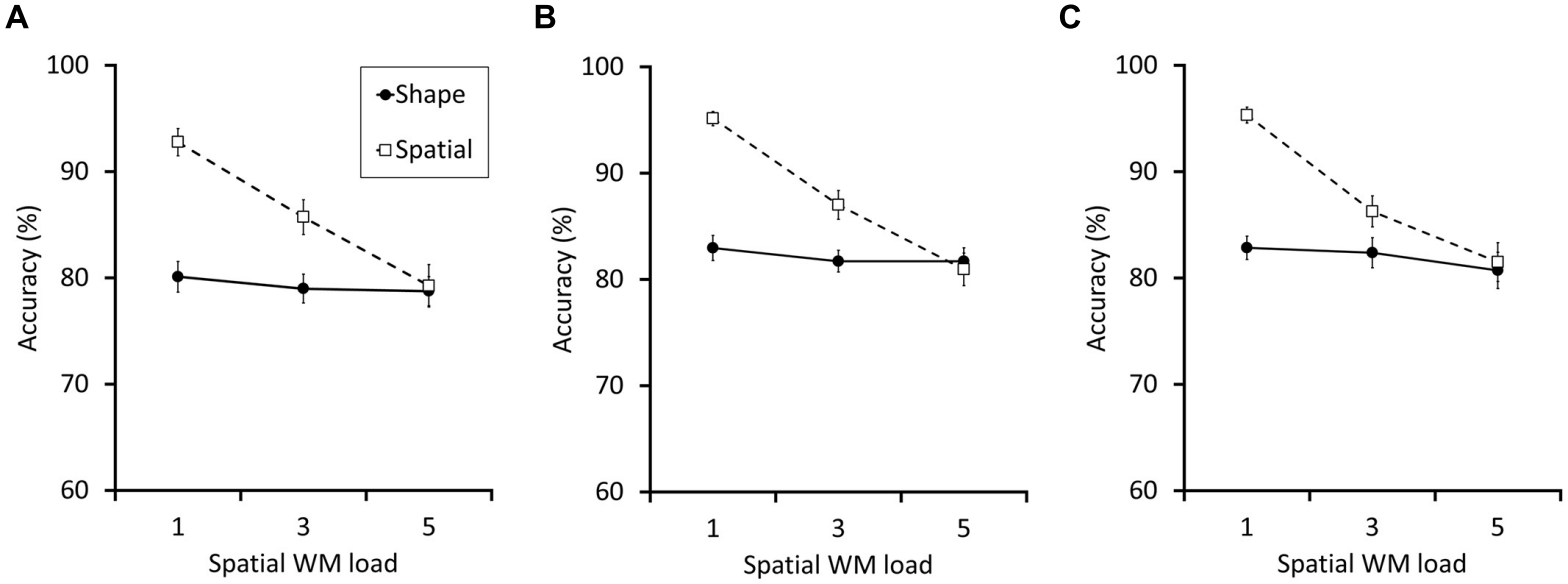
**The results for Experiments 1a (A), 1b (B), and 1c (C)**. The broken lines with empty squares and the solid lines with filled circles indicate the accuracy (% correct) of the spatial and the shape WM test trials, respectively. In all experiments, as the spatial WM load size increased, the performance of the spatial WM clearly decreased; the shape WM performance were, however, almost constant. The error bars indicate the SEM.

The results of Experiment 1a showed no evidence of interference between shape and spatial WM. Whereas higher spatial WM load significantly impaired the spatial WM score, it did not affect the shape WM performance (**Figure 2A**). We conducted a within-subject ANOVA on the accuracy data with the two factors, spatial WM load size (1, 3, or 5) and test type (spatial or shape). In addition to a significant main effects of spatial WM load size and test type [*F*(2,58) = 25.63, *p* < 0.001, $\eta_{p}^{2}$ = 0.47, *F*(1,29) = 24.03, 24.03, *p* < 0.001, $\eta_{p}^{2}$ = 0.45, respectively), the interaction between the two factors was also significant [*F*(2,58) = 16.79 *p* < 0.001, $\eta_{p}^{2}$ = 0.37]. *Post hoc* analyses showed that, as spatial WM load increased, accuracy in the spatial WM task decreased significantly (92.8, 85.7, and 79.3% for the load 1, 3, and 5, respectively; for each difference, *p*s < 0.001), but shape WM performance did not (80.1, 79.0, and 78.8% for the load 1, 3, and 5, respectively. *p*s > 0.995 for the difference between each load size). Finally, note that the insensitivity of shape WM score to the change of spatial WM load was apparently not due to a floor effect, because the mean performance in all conditions (around 80%) was far better than what would have been expected based on random guesses (50%).

### Experiment 1b

The procedure of Experiment 1b was almost the same as Experiment 1a, except that it adopted an alternative way to present the spatial cue sequence. In this experiment, the presentation interval was fixed to 1,500 ms (**Figure 1B**). This procedure eliminated the influence of the difference of interval length between conditions, and made it possible to test more precisely whether the consumption of spatial WM capacity disrupted the shape WM processing.

#### Method

##### Participants

Thirty-three volunteers (male: 18; female: 15; mean age: 19.53 years old, SD: 1.76) participated. They provided informed consent before the experiment and were compensated monetarily.

##### Stimuli and procedures

The procedure of Experiment 1b was different from Experiment 1a only in the way spatial WM cues were presented. The total duration of spatial cue presentation was fixed to 1,500 ms regardless of the load size. The presentation period was divided into five time slots, each of which lasted 300 ms. The appropriate number of slots (1, 3, or 5) was randomly chosen according to the load condition, and spatial memory cues (white dots) were presented at the selected slots (**Figure 1B**).

### Results and Discussion

Replicating Experiments 1a,b again showed an absence of interference between spatial and shape WM. We conducted a within-subject ANOVA on accuracy with the two factors spatial WM load size (1, 3, or 5) and test type (spatial or shape; **Figure 2B**). The main effects of spatial WM load size and test type were significant [*F*(2,64) = 50.10, *p* < 0.001, $\eta_{p}^{2}$ = 0.61,* F*(1,32) = 29.49, *p* < 0.001, $\eta_{p}^{2}$ = 0.48, respectively). In addition, the interaction between the two factors was also significant [*F*(2,64) = 23.29, *p* < 0.001, $\eta_{p}^{2}$ = 0.42]. *Post hoc* analyses showed that, as spatial WM load increased, the accuracy in the spatial WM task decreased significantly (95.1, 87.1, and 81.0% for load 1, 3, and 5, respectively, for each difference, *p*s < 0.001). By contrast, the accuracy did not decrease in the shape WM task (82.9, 81.7, and 81.7%, for load 1, 3, and 5, respectively. *p* = 0.80, *p* = 0.83, and *p*s > 0.995 for the difference between load 1–3, 1–5, and 3–5, respectively).

### Experiment 1c

In contrast to the first two experiments, spatial location cues were presented simultaneously for 500 ms in Experiment 1c (**Figure 1C**). The rest of the procedure remains exactly the same.

#### Method

##### Participants

Twenty-one volunteers (male: 8; female: 13; mean age: 21.0 years old, SD: 3.28) participated. They provided informed consent before the experiment and were compensated monetarily.

##### Stimuli and procedures

The only change from the procedures of Experiments 1a,b to Experiment1c was that the spatial WM cues were presented simultaneously for 500 ms (**Figure 1C**). The rest of the procedure remained exactly the same as in the two preceding experiments.

### Results and Discussion

The results of Experiment 1c proved that the cue presentation method is not the critical factor determining the between-domain WM interference (**Figure 2C**). Again, a within-subject ANOVA on accuracy was conducted with the two factors spatial WM load size (1, 3, or 5) and test type (spatial or shape; **Figure 2C**). Significant main effects of spatial WM load size and test type were confirmed [*F*(2,40) = 30.06, *p* < 0.001, $\eta_{p}^{2}$ = 0.60, *F*(1,20) = 33.15, *p* < 0.001, $\eta_{p}^{2}$ = 0.62, respectively). Critically, the interaction between the two factors reached significance [*F*(2,40) = 14.60, *p* < 0.001, $\eta_{p}^{2}$= 0.42. *Post hoc* analyses also showed the same results as the preceding two experiments. Spatial WM load increase significantly impaired the spatial WM task accuracy (95.3, 86.3, and 81.5% for load 1, 3, and 5, respectively, for the comparison between load 1–3, 1–5, *p*s < 0.001 and 3–5, *p* = 0.008). The accuracy of shape WM task, however, was not affected by the load manipulation (82.9%, 82.4%, and 80.7%, for load 1, 3, and 5, respectively. *p* > 0.995, *p* = 0.72, and *p* = 0.932 for the difference between load 1–3, 1–5, and 3–5, respectively).

In sum, Experiments 1a,b,c collectively provided results that were inconsistent with those of Wood (2011), showing no interference between shape and spatial WM performance and therefore suggesting that the storage resource was not shared between the two WM domains. In addition, since the data showed the same trend across the three experiments regardless of the spatial WM cue presentation method, the simultaneous presentation of spatial WM cues adopted in Wood (2011) was not the cause of the discrepancy.

## Experiment 2

The objective of Experiment 2 was to examine another possible confounding factor, namely the effect of task order, which was fixed (i.e., spatial then shape) in both our and Wood’s (2011) previous experiments. We simply tested whether the same pattern of results as in Experiments 1a,b,c could be observed even when the task order was reversed (i.e., shape then spatial).

### Method

#### Participants

Twenty-five volunteers (male: 9; female: 16; mean age: 21.40 years old, SD: 3.50) participated. They provided informed consent before the experiment and were compensated monetarily.

#### Stimuli and procedures

**FIGURE 3:**
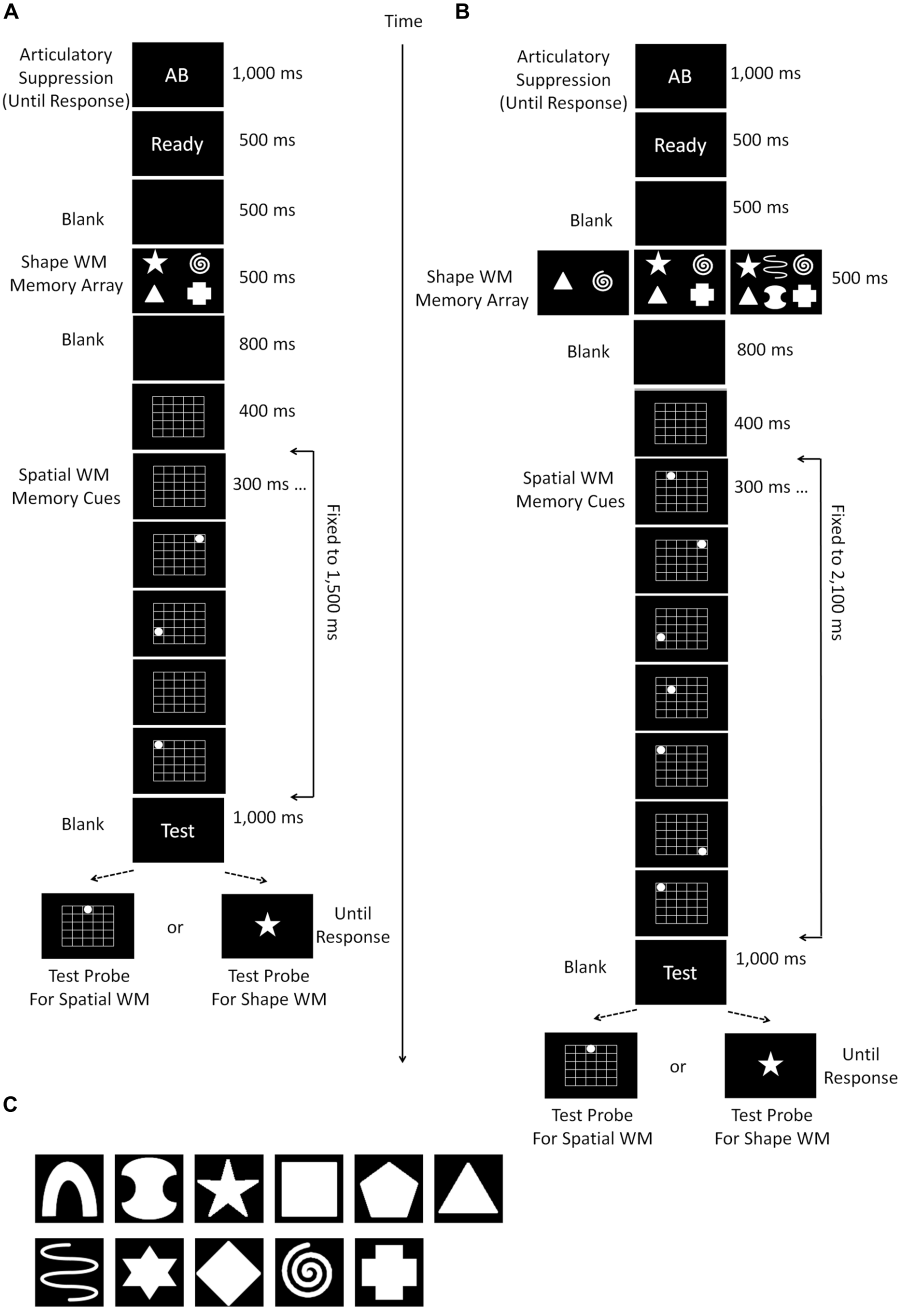
**Schematic diagrams of the experimental procedure used in Experiments 2 (A) and 3 (B)**. Experiment 2 was exactly the same as Experiment 1b except that the task order was reversed. In Experiment 3, the load size of shape WM was manipulated (2, 4, and 6) instead of the spatial WM (fixed to 7). **(C)** Shows the shape stimuli which were used for the shape WM task in Experiment 3.

The procedure of Experiment 2 was identical to that of Experiment 1b except that the order of the spatial and shape WM item presentation was reversed (**Figure 3A**FIG).

### Results and Discussion

**FIGURE 4:**
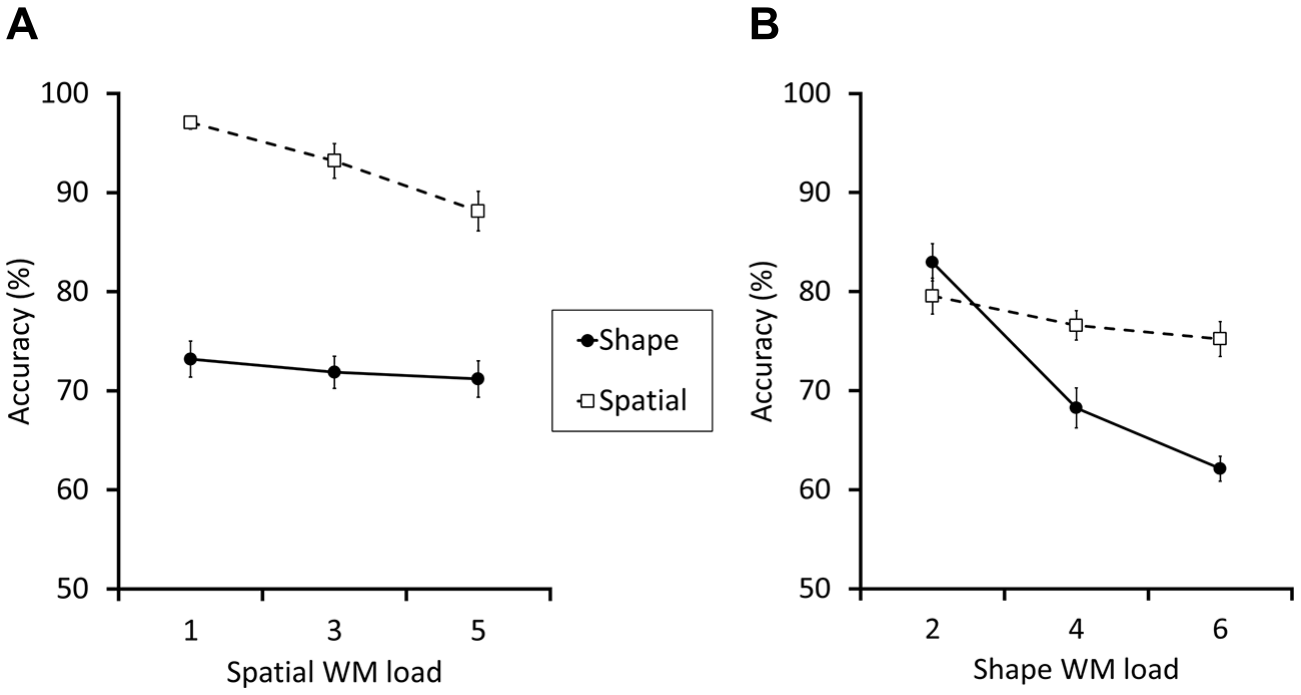
**The results for Experiments 2 (A) and 3 (B)**. The broken lines with empty squares and the solid lines with filled circles indicate the accuracy (% correct) of the spatial and the shape WM test trials, respectively. In Experiment 2, although the spatial WM accuracy apparently diminished along with the increment of load size, the shape WM did not. In contrast, the results of Experiment 3 indicated that the shape WM performance sharply fell as the load size increased, whereas the spatial WM did not. The error bars indicate the SEM.

Experiment 2 replicated the absence of interference between shape and spatial WM systems, regardless of the order of shape and spatial WM task assignments (**Figure 4A**). Again, we conducted a within-subject ANOVA with the two factors spatial WM load size (1, 3, or 5) and test type (spatial or shape). The main effect of load size, *F* (2,48) = 12.63, *p* < 0.001, $\eta_{p}^{2}$ = 0.35, and test type, *F*(1,24) = 127.54, *p* < 0.001, $\eta_{p}^{2}$ = 0.84 were both significant. Importantly, the interaction between the two factors was also significant, *F* (2,48) = 5.69, *p* = 0.006, $\eta_{p}^{2}$ = 0.19. *Post hoc* analyses showed that, as spatial load size increased, the spatial WM task became difficult (accuracy 97.0, 93.2, and 88.2% for load 1, 3, and 5, respectively; each difference, except between load 1 and 3, was significant, *p*s < 0.001), whereas there was no significant difference in the shape WM task (73.2, 72.0, and 71.2%, for load 1, 3, and 5, respectively. *p* = 0.98, *p* = 0.27, and *p* > 0.995 for the each difference between load 1–3, 1–5, and 3–5, respectively).

Moreover, the data suggested that the inversion of task order made the spatial WM task much easier compared to the previous two experiments, probably due to a shorter retention interval. In addition, there might have been a ceiling effect especially in the load 1 and 3 conditions. Thus, the smaller effect size of the interaction observed in the current experiment compared to Experiment 1 might be a mere consequence of this ceiling effect.

## Experiment 3

In Experiment 3, we manipulated the* shape*, but not the *spatial* WM load size. The load size for shape WM in the previous experiments in the current study was always set to 4. Thus, one might argue that an alternative interpretation for the results of Experiments 1a,b,c, and 2 would be that the shape WM impairment was absent because the shape WM reservoir was not completely occupied by the shape items, and therefore there was still room for spatial WM. This account, however, seems unlikely because many studies have proved that a load of four items fills up shape WM capacity quite sufficiently (e.g., Cowan, 2000). Nevertheless, we examined this alternative hypothesis, directly manipulating the shape WM load to confirm that there remained no extra space for additional spatial WM.

In addition, the results of Experiment 2 suggested that the shape-first, location-second task order apparently made the spatial WM task easier than in the previous two experiments, and we suspected that it might have produced a ceiling effect (see **Figure 4A**). This would become quite problematic in Experiment 3, because the aim of this experiment was to test the effect of shape WM load on spatial WM performance, and therefore the latter should remain sufficiently sensitive to the effect of interference. Thus, in Experiment 3, we employed the same settings as in Experiment 2 but increased the maximum item number for spatial WM from 5 to 7.

### Method

#### Participants

Twenty-two volunteers (male: 9; female: 13; mean age: 21.55 years old, SD: 3.51) participated. They provided informed consent before the experiment and were compensated monetarily.

#### Stimuli and procedures

The procedure of Experiment 3 was entirely the same as Experiment 2, except for the load size manipulation (**Figure 3B**). The shape WM load changed among 2, 4, and 6, and that for spatial WM was always fixed to 7. In previous experiments, the shapes were selected from seven items, but we added four more alternatives in Experiment 4 (i.e., 11 in total; see **Figure 3C**), because the maximum load size now increased to 6. When the load size was 4, the locations of presentation on the screen were the same as the previous experiments. When it was 2, the two shapes were presented at locations 5∘ horizontally right or left from the central fixation. Finally, when it was 6, the locations of four shapes were the corners of a width 13.1∘ × length 6.1∘ transparent rectangle appearing on the center of the screen, and the remaining two shapes were presented at the midpoint of the top and bottom sides of the rectangle (i.e., vertically 3.05∘ up and down from the fixation).

### Results and Discussion

Experiment 3 confirmed again the absence of the shape-location interference in WM regardless of which load size was manipulated (**Figure 4B**). We conducted a within-subject ANOVA on the accuracy, with the two factors shape WM load size (2, 4, or 6) and test type (spatial or shape). In addition to the significant main effects of shape WM load size and test type, [*F*(2,42) = 49.14, *p* < 0.001, $\eta_{p}^{2}$ = 0.70, *F*(1,21) = 9.64, *p* = 0.005, $\eta_{p}^{2}$ = 0.31, respectively), the interaction between the two factors reached significance, [*F*(2,42) = 25.36, *p* < 0.001, $\eta_{p}^{2}$ = 0.55. *Post hoc* analyses showed that, as the shape load size increased, the shape WM performance was more and more impaired (83.0, 68.1, and 62.1% for load 2, 4, and 6, respectively; for the difference between load 4–6: *p* = 0.01 and for others: *p*s < 0.001), whereas there was no significant difference in spatial WM performance (79.5, 76.6, and 75.2%, for load 2, 4, and 6, respectively. *p* = 0.33, *p* = 0.20, and *p* = 0.98 for the each difference between load 2–4, 2–6, and 4–6, respectively).

Thus, our data so far suggested no interference between the two WM systems, being inconsistent with the findings in Wood (2011). Importantly, however, there was a small, but consistent tendency for the change in the WM load to slightly impair the other, load-unrelated WM performance (i.e., shape WM task in Experiments 1 to 3, and spatial WM in Experiment 4; see **Figure 2A–C** and **4A,B**), even though they did not reach statistical significance in each separate experiment. Since the trend was always in the same direction, we suspected that these null results might have been simply due to statistically underpowered designs. Therefore, we additionally conducted a mixed 4 × 3 × 2 ANOVA on the pooled data across Experiments 1a,b,c, and 2. Experiment 3 was excluded because it adopted different ways of load manipulation. Data from 109 participants were included. The factors were experiment (i.e., Experiments 1a,b,c, or 2; between-subject), spatial WM load (1, 3, or 5; within-subject) and test type (spatial or shape; within-subject). This additional analysis revealed small, but statistically reliable between-domain interference. As in the separate analyses, a significant interaction between spatial WM load and test type was found [*F*(1.87,196.71) = 54.97, *p* < 0.001, $\eta_{p}^{2}$ = 0.34]. Also, *post hoc* analyses again showed a decrement of spatial WM as a function of the spatial WM load increase (95.1, 88.1, and 82.5% for load 1, 3, and 5, respectively. *p*s < 0.001 for each difference between the load sizes). In addition and most critically, the shape WM performance showed a statistically significant change between load 1 and 5 conditions (79.8, 78.7, and 78.1% for load 1, 3, and 5, respectively; *p* = 0.27, *d* = 0.16, *p* = 0.045, *d* = 0.24, and *p* > 0.995, *d* = 0.086, for the difference between load 1–3, 1–5, and 3–5, respectively). Thus, there was a small but significant performance impairment in the load-unrelated task, suggesting that spatial and shape WM share, if only to a small extent, a part of their storage systems.

## Experiment 4

In Experiment 4, we examined the robustness of the small interference effect observed in the abovementioned omnibus analysis. This time we used a design which followed that of Wood (2011) more precisely. The original study differed from our previous experiments in terms of that (1) it included zero load conditions, (2) it manipulated spatial and shape WM loads within the same experiment, and most critically, (3) it calculated a measure of interference called “combined dual-task interference” with the following equation;

Combined dual-task interference = [(% correct on color/shape/object memory task when performed alone) – (% correct on color/shape/object memory task when performed concurrently with spatial memory task)] + [(% correct on spatial memory task when performed alone) – (% correct on spatial memory task when performed concurrently with color/shape/object memory task)].

We adopted all these factors in Experiment 4. The only difference from the original study was the way to present spatial WM cues, which were shown simultaneously in the original but sequentially in the current experiment. Note that our Experiment 1 already showed that this difference was irrelevant for the results. We collected data from 90 participants to achieve a sufficiently high statistical power to detect any subtle effects. Assuming the abovementioned combined dual-task interference as a key measurement, a sample size of 90 was calculated to be sufficient to detect a relatively small effect size (assuming Cohen’s *d* = 0.3) with 80% statistical power in a two-tailed paired *t*-test.

### Method

#### Participants

Ninety-five volunteers were recruited (male: 53; female: 42; mean age: 24.9 years old, SD: 7.63), but data from five participants were discarded because they showed performance below the chance level (50%) in one of the conditions. Consequently, data of 90 participants were used. They provided informed consent before the experiment and were compensated monetarily.

#### Stimuli and procedures

**FIGURE 5:**
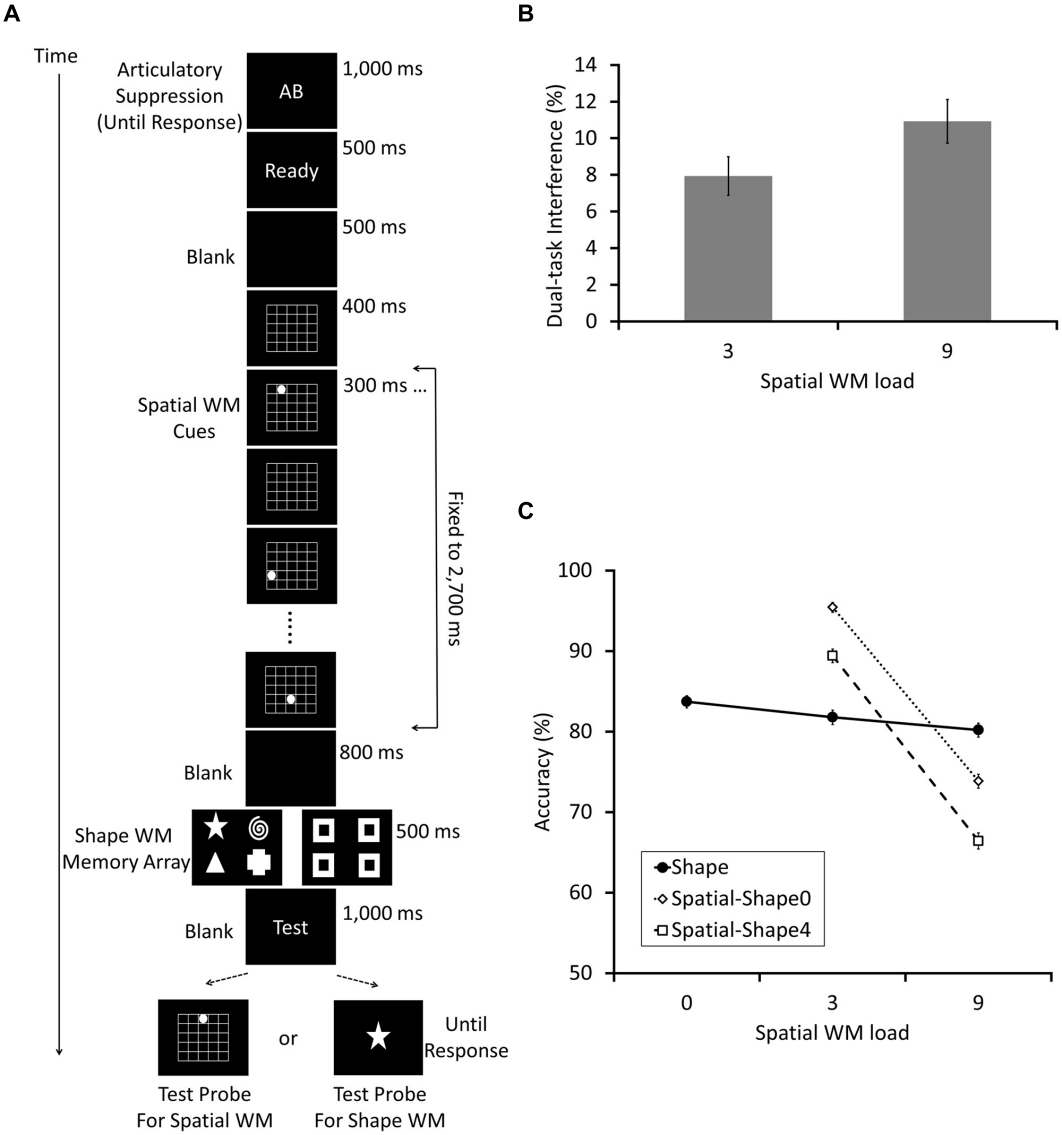
**The experimental procedure (A) and results (B,C) for Experiment 4, which was an exact replication of Experiment 2 of Wood (2011), except that the spatial cue presentation was conducted sequentially instead of simultaneously**. The load size of spatial WM randomly changed from 0, 3, to 5, and that of the shape were also manipulated (0 and 4) randomly. **(B)** Depicts the “combined dual task interference” scores, which were calculated following Wood (2011; see text for the details), as a function of spatial WM load. On the other hand, **(C)** shows the whole structure of the data, as we did in other experiments of the current study. In **(C)**, the dotted line with empty diamonds and the broken line with empty squares show the accuracy (%) correct of the spatial WM test trials when the shape WM load size was 0 and 4, respectively. The solid line with filled circles indicate the accuracy of the shape WM test trials. **(B,C)** Illustrate small but significant between-domain interference.

Following Experiment 2 of Wood (2011), we manipulated both spatial and shape WM load within experiment; the spatial WM load was set to 0, 3, or 9 locations and shape load was 0 or 4. The basic design was similar to Experiment 1b except that the total time period for spatial cue presentation was fixed to 2,700 ms. The period was divided into nine slots, each of which lasted 300 ms. The appropriate number of slots (0, 3, or 9) was randomly chosen according to the load condition, and spatial memory cues (white dots) were presented at selected slots (**Figure 5A**). When the shape load was 0, four “filler” objects were presented to equalize the perceptual demand across conditions. When spatial load was 0, only the grid was presented with no cues. The rest of the procedure remained exactly the same as Experiment 1b.

### Results and Discussion

We firstly analyzed our data following the methods described in Wood (2011). In sum, we successfully replicated most of the results reported in the study. First, a within-subject ANOVA was conducted for shape WM performance and showed a small but significant main effect of the spatial WM load increase [*F*(2,178) = 9.36, *p* < 0.001, $\eta_{p}^{2}$ = 0.095]. Second, another ANOVA was done for the spatial WM scores with the factors spatial (3 and 9) and shape WM load size (0 and 4), and revealed significant main effects of spatial [*F*(1,89) = 136.90, *p* < 0.001, $\eta_{p}^{2}$ = 0.61) and shape WM load [*F*(1,89) = 977.07, *p* < 0.001, $\eta_{p}^{2}$ = 0.92). We only failed to replicate an interaction between these two load factors [*F*(1,89) = 1.76, *p* = 0.19, $\eta_{p}^{2}$ = 0.019], which had been significant in the original study. These results are depicted in **Figure 5C** together. Third, the “combined dual-task interference” was calculated following the equation introduced in the Introduction section of the current article, and data was compared between 3 and 9 spatial WM load sizes using a two-tailed paired *t*-test (**Figure 5B**). A small but significant difference was found [*t*(89) = 2.31, *p* = 0.023; Cohen’s *d* = 0.24), which was also a successful replication of Wood (2011). Thus, the results of Experiment 4 demonstrated the robustness of the between-domain interference, confirming the report of Wood (2011). In addition, the effect size was very small, suggesting that separate analyses in Experiments 1–3 did not have sufficient power to detect it.

## General Discussion

The objective of the current study was to investigate the validity of the hypothesis that visual and spatial WM have two independent, separated storage systems. For this aim, we re-examined the results of the shape-location dual task experiment reported in Wood (2011), which provided putative evidence against the hypothesis. We examined several possibly confounding factors observed in the original study to account for the discrepancy. We tested whether the cue presentation procedure (Experiments 1a,b,c), order of tasks (Experiment 2), and the type of WM that was manipulated (Experiment 3) affected the interference effect. None of these factors had impact on the results, excluding the possibility that the results of Wood (2011) were confounded with some uncontrolled factors. However, we also failed to replicate the results of Wood (2011) in all experiments. When each experiment was separately analyzed, we found no evidence of shape-location interference. We found, however, an insignificant, but consistent trend of mean accuracy impairment in the load-unrelated WM tasks, which suggested the possibility that the experiments had too little power to detect the target effect. Therefore, we firstly re-analyzed our data combining those from Experiments 1a,b,c and 2, and obtained a statistically significant effect in the load-unrelated task. Moreover, an additional, direct replication of Wood (2011) with a sufficiently large data set (*N* = 90) confirmed the effect again, suggesting that the interference truly exists, but is so small that it could not be detected in separate experiments due to low statistical power.

Then, the critical question is how we should interpret these results in the context of the visuo-spatial WM separation hypothesis. The first issue of concern is which behavioral indices should be examined in order to correctly quantify the interference. Although we focused on the difference between non-zero load conditions throughout the current study (e.g., between the load 3 and 9 conditions in Experiment 4), another candidate would be the comparison between the zero and non-zero load conditions. We believe, however, that this is not an appropriate index to assess the dual task interference, because it possibly reflects an increase of general cognitive demand that is not specifically related to WM capacities.

In fact, several previous studies, which examined whether WM and visual search share the same limited resource, have used the difference between non-zero load conditions as an index to assess capacity sharing (Woodman et al., 2001; Oh and Kim, 2004; Woodman and Luck, 2004). For example, Woodman et al. (2001) discussed this problem explicitly, arguing that it is the steepness of the search slope, which is expressed as a function of the response time delay between non-zero load size conditions that should be affected by the capacity sharing. On the other hand, they argued that; if WM load impairs the processes that precede or follow the search process (e.g., response selection), this should produce a delay of response time in general, i.e., an increase of the intercept of the search function, but not a change in the steepness of the slope. In other words, even if the additional concurrent task produced a clear interference effect (i.e., an increase of the intercept), it should not be regarded as evidence that two tasks share a common cognitive resource.

To be sure, the data in Experiment 4 of the current study showed interference with large effect sizes between the zero and non-zero load conditions. The combined dual task interference scores were significantly larger than 0 (7.94%, *t*(89) = 7.55, *p* < 0.001, Cohen’s *d* = 0.80, and 10.93%, *t*(89) = 9.37, *p* < 0.001, Cohen’s *d* = 0.97, for load size 3 and 9, respectively). These results indicated that performing the dual task was more difficult than single tasks. However, we did not interpret these results as evidence suggesting capacity sharing between the two WM domains. Instead we focused on the difference between non-zero load conditions as an appropriate index of interference, the size of which was quite small across the experiments in the current study.

The second issue to consider is whether the performance interference itself, even if significantly detected between non-zero conditions, demonstrates an overlap of storage capacity between spatial and shape WM. This is not a straightforward question because the same result might have been obtained due to an increase of non-specific task difficulty that is not specific to WM storing. For example, Woodman et al. (2001) reported that the visual WM performance was generally deteriorated in a dual task paradigm, because of non-specific masking or interruption of WM items triggered by the simple stimulus presentation in the alternative task.

**FIGURE 6:**
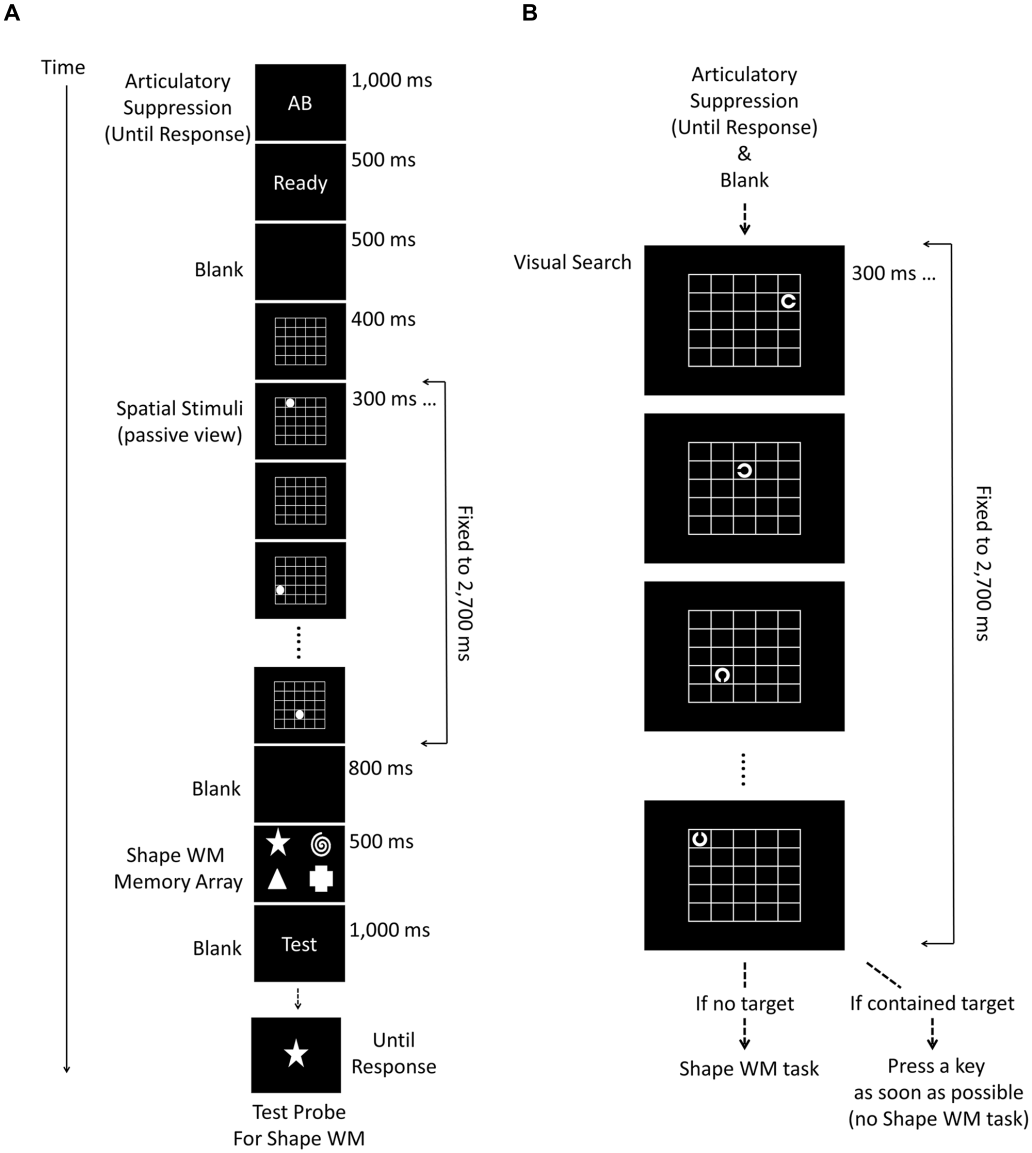
**Schematic diagrams of the experimental procedure used in additional preliminary experiments explained in Section “General Discussion.”** The experiment that examined whether perceptual masking caused the interference effect is shown in **(A)**, and the experiment that examined attentional processes in **(B)** (see General Discussion for details).

**FIGURE 7:**
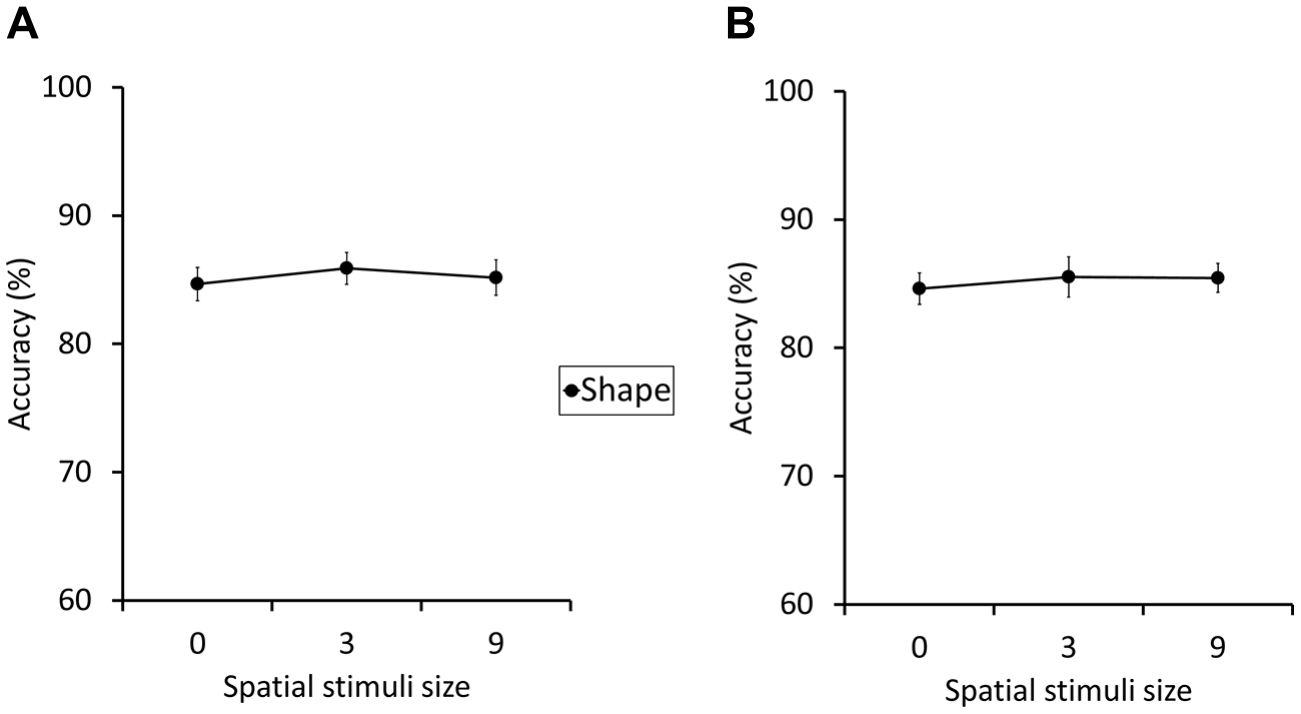
**The results of additional preliminary experiments discussed in Section “General Discussion,” depicting the accuracy (% correct) of the shape WM performance (see General Discussion for details). (A)** The perceptual masking experiment (see **Figure 6A**), and **(B)** the visual search experiment (see **Figure 6B**). The error bars indicate the SEM.

To obtain preliminary insights on this issue, we conducted two additional experiments with small data sets (*N* = 30 and 21, respectively). The first experiment tested whether simple presentations of location cue, without posing any spatial WM task, would cause an impairment of the concurrent shape WM task. The setting of Experiment 1b was adopted with the following modifications. Zero, three, or nine spatial location cues were presented, but spatial WM task was never required (**Figure 6A**). The results showed no statistically significant interference. Moreover, the mean accuracy even improved from load zero to three and nine conditions (84.7, 85.9, and 85.2%, respectively; **Figure 7A**). The second additional experiment examined whether the interference could be caused by attentional processes not involving WM storing. Participants were required to conduct a visual search task (**Figure 6B**), and asked to press a key if they found the target, after all search items had appeared (i.e., when the spatial grid disappeared). Search items were Landolt rings and the one that had an open part on the upper side was designated as target, which appeared randomly once in seven trials. Set size was manipulated among zero, three, and nine. The rest of the settings were the same as Experiment 1b. Results showed no significant effect of array size. In addition, a trend of shape WM improvement was observed again as the array size increased (84.8, 85.4, and 85.5% for array size 0, 3, and 9, respectively; **Figure 7B**).

Although the small sample sizes did not allow us to draw definite conclusions, the present pattern of results, i.e., improvement of mean accuracy score as the numbers of location cues increased, had never been observed in our previous experiments, where the genuine spatial WM task was required. Thus, we speculated that the mere perceptual masking and attentional processes were probably not the cause of the between-task interference, and therefore the effect was possibly triggered by some processes related to WM storing, suggesting that the two WM domains have an overlapping storage system.

However, it is also important to point out that the size of the interference was quite small across Experiments 1–4, and this observation makes us hesitate to conclude that the two systems fully share their capacities. Presumably, the most straightforward way for measuring the interference between two storage systems is to test whether the maximum number of items that can be remembered remains the same, regardless of which item dimension is assigned to memorize (Zhang et al., 2012). If two (or more) WM domains have a fully interdependent storage system, then the maximum capacity should be unchanged and limited to the same amount, regardless of which WM load was manipulated. Woodman et al. (unpublished data) reported a good example of such examination in the visual domain (cited in Zhang et al., 2012). They tested whether color and orientation WM shared a common system by comparing the performance between when participants had to remember six color or orientation items and when they were required to memorize three from each category. The results showed no significant difference between two conditions, suggesting that the two memory capacities overlap to a significant degree. Assuming that this type of result is the gold standard to demonstrate a full capacity overlap between WM domains, the small effect size we obtained in the current study seemed insufficient to state that spatial and shape WM systems fully shared their storage systems.

This interpretation might solve some of the disparities observed in previous studies, including Wood (2011). As we have already discussed in Introduction, there has been a profound inconsistency among the previous studies on this issue. On the one hand, some dual task studies supported the distinction between visual and spatial cognitive systems by showing the absence of interference between them (Logie and Marchetti, 1991; Tresch et al., 1993; Woodman et al., 2001; Woodman and Luck, 2004). On the other hand, other researchers proposed alternative hypotheses that conflicted with the idea of simple visuo-spatial separation (Jiang et al., 2000; Wheeler and Treisman, 2002). Finally, the results of Wood (2011) further deepened the problem, since none of the previous theories was consistent with the data. Possibly, one of the reasons for these inconsistent results was that some of the studies were simply underpowered to detect the between-domain interference, as shown in our Experiments 1–3.

In sum, we conclude that, whereas some parts of shape and spatial WM systems are clearly overlapping, their capacities are mostly independent from each other. Thus, our data support the visuo-spatial WM separation hypothesis proposed by Logie (1995). Moreover, our results were in line with the argument proposed by Wheeler and Treisman (2002). They argued that keeping spatial information is necessary for WM maintenance of conjunctive objects (e.g., “blue triangle”), but not for simple features (e.g., “blue” or “triangle”). However, further research is required to fully resolve the controversy. First, since the original visuo-spatial WM separation hypothesis maintains the independence of spatial WM capacity not only from the feature (e.g., shape) but also the object WM system (Logie, 1995), the object-spatial WM interference should also be examined. Second, Jiang et al. (2000) suggested that storing even simple features needs retention of their spatial relationship, and this seems to be incompatible with the current study. Jiang et al. (2000), however, utilized change detection tasks, the structure and processing of which could be significantly different from the single probe task we employed in the current study. Therefore, this task difference should also be investigated in future research.

Another important concern would be the neural substrates underpinning this phenomenon. The First candidate would be the prefrontal cortex. Using electrophysiological recordings of neural activity in macaque monkeys, Wilson et al. (1993) suggested that visual and spatial WM could be separately implemented in the ventro- and dorso-lateral areas of the prefrontal cortex. This hypothesis was further supported by human imaging studies (Courtney et al., 1996, 1998; cf. Mishkin et al., 1983). However, many other studies have provided evidence inconsistent with this simple dichotomy (Rao et al., 1997; Rushworth et al., 1997; Postle et al., 2000). For example, by utilizing lesion technique, Rushworth et al. (1997) found that the ventral prefrontal area, the inferior convexity in particular, was not important for WM processing. In addition, Rao et al. (1997) showed that the neural populations in the prefrontal area which correlated with visual or spatial WM could not be separated simply. They found that many neurons showed activity related to both visual and spatial WM maintenance. Finally, Postle et al. (2000) tried to examine the abovementioned separation at the neural level in humans, but failed to replicate previous studies. Thus, this possibility still remains highly controversial. In contrast, the distinction between ventral visual and dorsal spatial pathway in the visual cortex has been largely accepted among researchers (Mishkin et al., 1983). There has been, however, still no direct evidence to connect this to the separation between WM domains. Further research is clearly needed to shed light on this issue.

## Conflict of Interest Statement

The authors declare that the research was conducted in the absence of any commercial or financial relationships that could be construed as a potential conflict of interest.
